# Maternal and perinatal outcomes of oligohydramnios in late term and post term pregnancies at public hospitals in Ethiopia: a cross-sectional study

**DOI:** 10.1186/s12905-024-02952-0

**Published:** 2024-02-12

**Authors:** Matiyas Asrat Shiferaw, Ananya Solomon, Sintayehu Getachew, Wondimu Gudu

**Affiliations:** 1 Department of Obstetrics and Gynecology, Saint Paul’s Hospital Millennium Medical College, Addis Ababa, Ethiopia; 2 Department of Obstetrics and Gynecology, Yekatit 12 Hospital Medical College, Addis Ababa, Ethiopia; 3 Department of Obstetrics and Gynecology, Ras Desta Damtew Hospital, Addis Ababa, Ethiopia

**Keywords:** Oligohydramnios, Outcome, Late term pregnancy, Post term pregnancy

## Abstract

**Background:**

The prevalence of oligohydramnios ranges from 12 to 14% after 41 weeks to as high as 30% in post term pregnancies. Oligohydramnios poses a dilemma in the choice of mode of labor and delivery in a setup where there is lack of continuous electronic fetal monitoring during labor. The condition also puts the mother at risks of operative interventions and cesarean delivery. We aimed to asses the maternal and perinatal outcomes in pregnancies with oligohydramnios in late term and post term pregnancy in this study.

**Methods:**

A cross-sectional study was conducted among mothers with diagnosis of oligohydramnios after 40^+ 6^ weeks of gestation at four hospitals at four public hospitals in Addis Ababa, Ethiopia from May 1, 2021 to September 30, 2021. Data were collected using structured questionnaire. Logistic regression were performed to assess factors associated with the adverse maternal and perinatal outcomes.

**Results:**

From a total of 142 mothers with oligohydramnios in late term and post tem pregnancies, 40.8% delivered through cesarean section. Spontaneous labor and elective cesarean section were more likely to occurr in parous women (AOR 2.5, 95% CI 1.06–6.04, *p* = 0.04), but with less likely in those with secondary level education (AOR 0.13, 95% CI 0.02–0.74, *p* = 0.02). There was no statistically significant difference in adverse outcomes between those who had induction of labor and those who had either spontaneous labor or had elective cesarean section.

**Conclusions:**

The adverse maternal and perinatal outcomes in late term and post term pregnancies with oligohydramnios may not be different among different modes of delivery. Induction of labor can be safe in these particular group of women with intermittent auscultation with fetoscope in a setup where continuous electronic fetal monitoring is not readily available.

## Background

Oligohydramnios is term used to describe a state of decreased amniotic fluid defined as single deepest vertical pocket less than 2 centimeters and/or amniotic fluid index less than 5 centimeters on ultrasound [1]. The prevalence of oligohydramnios depends on the gestational age and ranges from 1 to 5% at term but it can go as high as 12–14% and 30% after 41 weeks and in post term pregnancies respectively [1–5]. A study from Ethiopia reported a prevalence of 2.3% at term [6].

Lack of amniotic fluid at term is thought to be associated with a number of adverse antepartum, intrapartum and perinatal outcomes. There is a greater risk for nonreactive nonstress tests, increased risk for labor inductions, fetal heart rate decelerations in labor, meconium stained amniotic fluid, cesarean delivery for fetal labor intolerance, increased risk of stillbirth, neonatal intensive care unit (NICU) admissions, low APGAR scores and neonatal deaths [7–9]. Because of these risks, when isolated oligohydramnios is noted at term, induction of labor has been routinely recommended. On the other hand, some studies have suggested that the risk of adverse perinatal outcomes of oligohydramnios is increased only when pregnancy advances beyond 41 weeks’ gestation or when complicated with intrauterine fetal growth restriction (IUGR) [10, 11].

Oligohydramnios puts the mother at risks of operative interventions and cesarean delivery [3, 4, 12, 13]. It is associated with increased risk of delivering via cesarean section (CS) primarily due to non reasuring fetal heart rate pattern in labor [2, 14, 15]. Studies show high rate of CS in both high and low income countries ranging from 42.0 to 83.6% [2–4, 6, 7, 16, 17].

There are some studies, on the other hand, that demonstrated oligohydramnios does not predict maternal and neonatal outcome. These studies suggested the need for increased pregnancy surveillance when oligohydramnios is detected. Otherwise, the mere presence of oligohydramnios cannot justify pregnancy interventions including induction or cesarean delivery [5, 13, 18]. In general there is no adequate evidence to optimize the management of women with oligohydramnios and hence has always been open for controversy.

At public hospitals in Ethiopia, there is scarcity of cardiotocography machines and not all mothers get continuous fetal monitoring with tracing during labor. Therefore, owing to the perceived inadequate intrapartum follow up and the increased rate of CS in labor in those who are induced, there is a trend to lower a threshold to do elective CS in cases of oligohydramnios.

There is a dilemma in management of oligohydramnios especially in set ups lacking continuous intrapartum fetal monitoring [5]. The CS rates are rising due to intrapartum complication and high rate of perinatal morbidity and mortality associated with oligohydramnios. The choice of decision between vaginal and caesarean delivery should be well balanced so that unnecessary maternal morbidity is avoided and perinatal morbidity and mortality are prevented [5, 16].

Context specific appreciation of factors related to poor outcome in oligohydramnios could help stratify management of these mothers. It can also serve as a baseline local data against which mothers could be advised and counseled about the degree of maternal and perinatal morbidity as well as mortality associated with the condition.

There is paucity of published work on factors affecting maternal and perinatal outcome of oligohydramnios in late term and post term pregnancy in Sub-Saharan countries, includung Ethiopia. Most of the studies done elsewhere in the world included pregnancies that are either preterm or term at diagnosis of oligohydramnios. To our knowledge there is no published study in outcome of oligohydramnios specifically in late term and post term pregnancies. This study was designed to assess [1] the maternal and perinatal outcome of pregnancies complicated with oligohydramnios in late term and post term and [2] factors affecting the outcomes.

## Materials and methods

### Study settting and period

The study was conducted at four public hospitals in Addis Ababa, Ethiopia from May 1, 2021 to September 30, 2021. The hospitals were Saint Paul’s Hospital, Menelik II hospital, Ras Desta Damtew Hospital and Yekatit 12 Hospital and were located in Addis Ababa, Ethiopia. Saint Paul’s Hospital, which was under St.Paul’s Hospital Millennium Medical College (SPHMMC), was one of the largest hospital providing a teritiary level services in Ethiopia. The hospital provides labor and delivery service to an average of 900 women monthly. The other hospitals were affiliates of SPHMMC and each had a mean monthly deliver of 400 women.

In the hospitals, induction of labor was the standard of practice in mothers who develop oligohydramnios beyond term. Mothers with unfavorable BISHOP were primed with Foley catheter. Induction of labor was contraindicated in the following circumstances: previous cesarean sections, breech presentation, and maternal bad obstetrics history.

### Study design

This was a cross-sectional study.

### Study population and eligibility criteria

The study was conducted among mothers with diagnosis of oligohydramnios after 40 + 6 weeks of gestation at four hospitals. We included women with singleton pregnancies who were diagnosed to have oligohydramnios by ultrasound after 41^+ 0^ weeks of gestation and before the onset of labor in the study. Gestational age was calculated from reliable date or early ultrasound done before 24^+ 0^ weeks of gestation. We excluded the women if there was premature rupture of membrane (PROM), congenital malformation of the fetus or intrauterine fetal death (IUFD) at presentation.

### Sample size determination

Sample size was calculated using single population proportion formula with 9.7% prevalence of composite adverse neonatal outcome in oligohydramnios from a previous study [19]. Assuming margin of error of 5% and 10% nonresponding rate, the estimated sample size was 149.

We recruited consecutive mothers presenting with oligohydramnios after 41 + 0 weeks of gestation. The sample size was allocated to the hospitals based on the number of deliveries in the previous three months before data collection using proportionate to population size sampling method.

### Data collection and data quality assurance

Structured questionnaire, which was developed by reviewing different literatures, was used for the study. The questionnaire was prepared in English, was translated to Amharic then translated back to English to check for consistency. The questionnaire was pretested with 5% of patients, which were excluded from the sampling frame later. Mothers who fulfill the inclusion criteria were enrolled for interview after receiving counseling from trained pregnancy advisors and signing a consent form. Eight trained midwives, who were not involved in the care for the patient, collected the data and four obstetrician and gynecologists supervised the data collection. Operational manual for the study, with detailed instruction to the data collectors, was prepared to assure a uniform standard to carrying out the study with good quality control.

### Study variables

We collected data on on participant’s age, place of residence, level of education, occupation, marital status, parity, antenatal care (ANC), HIV serostatus, antepartum maternal complication (APH (antepartum hemmhorrhage), preeclampsia, diabetes, chronic hypertension, thyroid disorders, anemia), presence of IUGR, APGAR scores, NICU admissions, perinatal death, non reasuring fetal heart rate pattern (NRFHRP) and mode of delivery.

### Operational definitions

The following operational definitions were used. Late term pregnancy was used if the gestational age between 41^+ 0^ week to 41^+ 6^ weeks. Post term pregnancy was defined if gestational age is greater than 41^+ 6^ weeks of pregnancy. Olighydramnios was considered if AFI ≤ 5 or SDVP ≤ 2. Women with hemoglobin less than 7 g per deciliter were considered to have anemia.

Composite adverse fetal outcome was defined if one of the following occurs: first minute APGAR less than seven, NICU admission, still birth or early neonatal death.

### Statistical analysis

The data was entered into Epi-info version 3.5.1 and transported to SPSS version 25 software packages for analysis. Frequencies and basic descriptive statistics were calculated for all variables, with correlations and Chi square statistics calculated for key variables. Bivariable analysis was carried out first to observe the crude association between independent and dependent variables. Those variables with *p*-values < 0.25 on bivariate analysis were then fitted into multivariable logistic regression analysis. Adjusted odds ratio (AOR) were calculated and those variables with *p*-value of 0.05 were considered to be significantly associates with the outcome of interest.

## Results

### Sociodemographic characterstics

A total of 142 women, who fulfilled the inclusion criteria and complete data, were included in the final analysis. Most of the study participants attended at least elementary school. The mean age of the participants was 26.95 (± 4.34) years. Majority were housewives (50.7%) and urban dwellers from Addis Ababa (76.8%). (Table 1)

**Table 1:** Demographic characteristics of women presented with the diagnosis of oligohydramnios in pregnancies beyond 41 weeks of gestation at selected hospitals in Addis Ababa from May 1, 2021 to September 30, 2021

	Frequency	Percent
**Age**		
≤ 19	1	0.7%
20–34	131	92.3%
≥ 35	10	7.0%
Total	142	100%
**Education**		
No formal education	17	12.0%
Elementary	53	37.3%
Secondary	42	29.6%
College or university	30	21.1%
Total	142	100%
**Occupation**		
Housewife	72	50.7%
Farmer	1	0.7%
Self employed	39	27.5%
Government employee	30	21.1%
Total	142	100%
**Place of living**		
Rural	33	23.2%
Urban	109	76.8%
Total	142	100%

### Obstetrics and medical profile of paricipants

The median number of child birth prior to the index pregnancy was 1 (± 1). Most of the gestational age of the pregnancies falls between 41 completed weeks and 41 weeks and 6 days. Anemia and hypertensive disorders of pregnancies were the most common antepartum maternal complications seen. (Table 2)

**Table 2:** Obstetrics and medical conditions of women presented with the diagnosis of oligohydramnios in pregnancies beyond 41 weeks of gestation at selected hospitals in Addis Ababa from May 1, 2021 to September 30, 2021

	Frequency	Percent
**Parity**		
0	68	47.9%
1–3	72	50.7%
≥ 4	2	1.4%
Total	142	100%
**Gestational age**		
41 weeks to 41 weeks 6 days	91	64.1%
≥ 42 weeks	51	35.9%
Total	142	100%
**Antenatal care**		
Three visit	6	4.2%
Four and above visit	136	95.8%
Total	142	100%
**Antepartum complication**		
DM	1	2.9%
Anemia	13	37.1%
Hypertensive disorders in pregnancy	11	31.4%
Abnormal placentation	5	14.3%
IUGR	3	8.6%
Asthma	1	2.9%
HIV	1	2.9%
Total	35	100%

### Labor and delivery conditions of participants

Labor was induced in three quarters of the women. All except two were primed with Foley catheter before induction with oxytocin. Labor started spontaneously in 25 (17.6%) of the women. 7% of the women underwent elective CS before the onset of labor. 58% of women with induced labor and 84% of those with spontaneous onset of labor delivered vaginally. Overall rate of CS was 40.8%. One women among the induced underwent laparotomy for iatrogenic uterine rupture. (Table 3)

**Table 3:** Labor and delivery conditions of women presented with the diagnosis of oligohydramnios in pregnancies beyond 41 weeks of gestation at selected hospitals in Addis Ababa from May 1, 2021 to September 30, 2021

	Frequency	Percent
**Labor onset**	25	17.6%
Spontaneous	25	17.6%
Induced	107	75.4%
Elective CS	10	7.0%
Total	142	100%
**Mode of delivery with spontaneous onset of labor**		
Vaginal	20	80%
emergency CS	4	16%
Operative vaginal delivery	1	4%
Total	25	100%
**Mode of delivery in induced**		
Vaginal	60	56.1%
Emergency CS	44	41.1%
Operative vaginal delivery	2	1.9%
Laparotomy for uterine rupture	1	0.9%
Total	107	100%
**Overall Mode of delivery**		
Vaginal	79	55.6%
Emergency CS	48	33.8%
Operative vaginal delivery	4	2.8%
Elective CS	10	7.0%
Laparotomy	1	0.7%
Total	142	100%

The commonest indication for emergency CS was non reassuring fetal heart rate pattern (NRFHRP) 38(79.2%). The two most common indications for elective CS were previous cesarean scar and macrosomia accounting for 40% and 30% of the elective cesarean sections respectively. (Table 4)

**Table 4:** Indication for cesarean section among women presented with the diagnosis of oligohydramnios in pregnancies beyond 41 weeks of gestation at selected hospitals in Addis Ababa from May 1, 2021 to September 30, 2021

	Frequency	Percent
**Indication for elective cesarean section**		
Oligohydramnios + breech presentation	2	1%
Oligohydramnios + previous CS Scar	4	40%
Oligohydramnios + Macrosomia	3	30%
Oligohydramnios + bad obstetric history	1	10%
Total	10	100%
**Indication for emergency Cesarean section**		
NRFHRP	38	79.2%
Previous CS scar + Prolonged latent	1	2.1%
GIII MSAF on induction	4	8.3%
Labor abnormality	1	2.1%
Failed induction	4	8.3%
Total	48	100%

### Maternal and perinatal outcomes

The mean birth weight was 3271 g. The male to female ratio was 1.1. The first minute APGAR was less than 7 in 11 (7.7%). 5% of the neonates were referred to NICU. Intrapartum and early neonatal death occurred in 3(2.1%). (Table 5)

**Table 5:** Perinatal outcome among women presented with the diagnosis of oligohydramnios in pregnancies beyond 41 weeks of gestation at selected hospitals in Addis Ababa from May 1, 2021 to September 30, 2021

	Frequency	Percent
**Sex**		
Male	75	52.8%
Female	67	47.2%
Total	142	100%
**1st minute APGAR**		
< 7	11	7.7%
≥ 7	131	92.3%
Total	142	100%
**Birth weight**		
< 2500	3	2.1%
2500–3999	127	89.4%
≥ 4000	12	8.5%
Total	142	100%
**NICU referral**		
Yes	7	4.9%
No	135	95.1%
Total	142	100%
**Intrapartum and early neonatal death**		
Stillbirth	1	33.3%
Early neonatal death	2	66.7%
Total	3	100%

The baseline characteristics of the mothers who had labor induction was not different from those with either spontaneous labor or elective CS except in terms of parity. Parous women have increased odds of elective CS and spontaneous labor than nulliparous women (AOR 2.5, 95% CI 1.06–6.04, *p* = 0.04) in multivariable regression. Those with secondary education were less likely to have elective CS and spontaneous labor than those who did not have formal education (AOR 0.13, 95% CI 0.02–0.74, *p* = 0.02) after controlling for place of living, occupation and parity in regression analysis. (Table 6)

**Table 6:** Characteristics and labor and delivery outcome for the induction and non-induction groups among women presented with the diagnosis of oligohydramnios in pregnancies beyond 41 weeks of gestation at selected hospitals in Addis Ababa from May 1, 2021 to September 30, 2021

	Induced labor (*N* = 107)	Spontaneous labor or elective CS (*N* = 35)	*P* value
**Age**			
< 35	100 (93.5%)	32 (91.4%)	0.71
≥ 35	7 (6.5%)	3 (8.6%)	
**Place of living**			
Rural	22 (20.6%)	11 (31.4%)	0.25
Urban	85 (79.4%)	24 (68.6%)	
**Education**			
No formal education	12 (11.2%)	5 (14.3%)	0.1
Primary	35 (32.7%)	18 (51.4%)	
Secondary	33 (30.8%)	9 (37.1%)	
College	27 (25.2%)	3 (14.3%)	
**Occupation**			
Housewife	56 (52.3%)	16 (45.7%)	0.12
Farmer	0	1 (2.9%)	
Self employed	26 (24.3%)	13 (%)	
Government employee	25 (23.4%)	5 (4%)	
**Parity**			
Nulliparous	57 (53.3%)	11 (31.4)	0.03
Parous	50 (46.7%)	24 (68.6%)	
**Gestational age**			
41 weeks to 41 weeks 6 days	66 (61.7%)	25 (71.4%)	0.31
≥ 42 weeks	41 (39.3%)	10 (29.6%)	
**Antepartum complication**	24 (22.4%)	10 (28.6%)	0.50
**1st minute APGAR < 7**	10 (9.3%)	1 (2.9%)	0.29
**Birth weight**			
< 2500	2 (1.9%)	1 (2.9%)	0.71
2500–3999	97 (90.7%)	70 (85.7%)	
≥ 4000	8 (7.5%)	4 (11.4%)	
**NICU referral**	6 (5.6%)	1 (2.9%)	0.51
**Intrapartum and early neonatal death**	3 (2.8%)	0	0.32
**Adverse perinatal outcome**	17 (15.9%)	1 (2.9%)	0.32
**Adverse maternal outcome**	6 (5.6%)	1 (2.9%)	0.51

Composite adverse maternal outcome was considered if the mothers had either post partum hemorrhage or uterine rupture or maternal death. There was no significant difference in terms of composite adverse maternal outcome whether labor was induced or not. (Tables 6 and 7)

**Table 7:** Characteristics, and labor and delivery outcome for the induced and spontaneous labor among women presented with the diagnosis of oligohydramnios in pregnancies beyond 41 weeks of gestation at selected hospitals in Addis Ababa from May 1, 2021 to September 30, 2021

	Induced labor (*N* = 107)	Spontaneous labor (*N* = 25)	*P* value
**Age**			
< 35	100 (93.5%)	23 (92%)	0.71
≥ 35	7 (6.5%)	2 (8%)	
**Place of living**			
Rural	22 (20.6%)	8 (32%)	0.29
Urban	85 (79.4%)	17 (68%)	
**Education**			
No formal education	12 (11.2%)	3 (12%)	0.02
Primary	35 (32.7%)	16 (64%)	
Secondary	33 (30.8%)	5 (20%)	
College	27 (25.2%)	1 (4%)	
**Occupation**			
Housewife	56 (52.3%)	14 (56%)	0.03
Farmer	0	1 (4%)	
Self employed	26 (24.3%)	9 (36%)	
Government employee	25 (23.4%)	1 (4%)	
**Parity**			
Nulliparous	57 (53.3%)	7 (28)	0.03
Parous	50 (46.7%)	18 (72%)	
**Gestational age**			
41 weeks to 41 weeks 6 days	66 (61.7%)	18 (72%)	0.37
≥ 42 weeks	41 (39.3%)	7 (28%)	
**Antepartum complication**	24 (22.4%)	8(32%)	0.31
**1st minute APGAR < 7**	10 (9.3%)	1 (4%)	0.69
**Birth weight**			
< 2500	2 (1.9%)	1 (4%)	0.31
2500–3999	97 (90.7%)	24 (96%)	
≥ 4000	8 (7.5%)	0	
**NICU referral**	6 (5.6%)	1 (4%)	0.75
**Intrapartum and early neonatal death**	3 (2.8%)	0	0.40
**Adverse perinatal outcome**	17 (15.9%)	1 (4%)	0.20
**Adverse maternal outcome**	6 (5.6%)	1 (4%)	0.75
**Mode of delivery**			
Vaginal delivery	62 (57.9%)	21 (84%)	0.02
Cesarean section	45 (42.1%)	4 (6%)	

After controlling for parity there was lower rate of CS in those with spontaneous labor than those with induced labor (AOR 0.31, 95% CI 0.10–0.96, *p*-value = 0.04).

Individual or composite adverse outcomes of women who had induced were not significantly different from those who had spontaneous labor and/or who had elective CS. (Tables 6 and 7)

## Discussion

We report on clinically well-characterized participants with oligohydramnios in late term and post term pregnancy treated in public hospitals. We had a wealth of data on the participants and this provided us a perspective on the maternal and perinatal outcomes of oligohydramnios in late term and post term pregnancy. To our knowledge, this is the first published study in a low resource setup where continuous electronic monitoring of the fetus during labor and delivery is not readily available. The overall rate of CS in our study population (40.8%) was high.The commonest indication for emergency CS among the laboring women was NRFHRP (79.2%). High rate of cesarean section was expected in mothers with oligohydramnios considering their risk of developing non reassuring fetal heart rate during labor [2, 14, 15].

There was significantly higher rate of emergency CS among the induced women than those with spontaneous onset of labor. This was also observed in previous observational studies comparing induction with spontaneous labor [20, 21]. This higher rate of CS can be because of failed induction and abnormal fetal heart beat patterns But we should note that there is lower rate of CS with induction compared with expectant management in late term pregnancies [22].

In our study there was no significant difference im maternal and perinatal outcomes between women who were induced and those who had either spontaneous labor or undergone elective CS. Most of the women in our study were primed with Foley catheter and induced. Midwives followed the women with intermittent auscultation using fetoscope during labor and delivery. However, in another study that included subjects with advanced maternal age, induction of labor was associated with an increased risk for composite adverse perinatal and maternal outcomes. Adverse outcomes was more strongly associated with Induction of labor in comparison with spontaneous onset of labor than increased maternal age was [23]. The finding of our study suggests that it may be safe to induce labor in late term and post term pregnancies with oligohydramnios in low resource setups where continuous electronic fetal heart rate monitoring is not readily available. But this should be interpreted cautiously as the study design was not powered enough to make any cause effect associations.

Although there was one case of uterine rupture among the women induced, there was no significant difference in adverse maternal outcome between those who induced and those who had either spontaneous labor or had elective CS. The uterine rupture occurred in a para 3 women with out previous cesarean scar after being primed with Foley catheter and induction of labor. Spontaneous onset and induced labors have similar risk of uterine rupture if we account for the duration of labor [24]. Therefore, induction of labor in late term and post term pregnancies can be considered safe for the mothers.

The study had some limitations. The cross-sectional nature of the study design will render the strength of any cause and effect associations weak. The data was collected from a limited number of hospital population. This might limit generalizability of the results to women who will have labor and delivery management at lower health facilities. In addition, most of the study participants were from urban area and this makes our inference limited to urban population.

## Conclusion

The rate of CS among women with oligohydramnios in late term and post term pregnancies was high. However, adverse maternal and perinatal outcomes after induction of labor were not significantly different from those with either spontaneous labor or elective CS. Mode of labor and deliver did not affect the adverse maternal and perinatal outcome. Induction of labor with intermittent fetoscopic fetal hear beat monitoring looked safe in these particular group of women even in the absence of continuous electronic fetal monitoring. But this should be substantiated with well designed future studies.

## Data Availability

All data generated and/or analyzed are included in this article.

## Abbreviations

ANC: Antenatal care
AFI: Amniotic fluid index
AOR: Adjusted odds ratio
APH: Antepartum hemmhorrhage
CI: Confidence Interval
CS: Cesarean section
IUFD: Intrauterine fetal death
IUGR: Intrauterine fetal growth restriction
NICU: Neonatal intensive care unit
NRFHRP: Non reasuring fetal heart rate pattern
PROM: Premature rupture of membrane
SDVP: Single deepest vertical pocket
SPHMMC: St.Paul’s Hospital Millennium Medical College
